# Boy With Facial Pain

**DOI:** 10.1016/j.acepjo.2026.100496

**Published:** 2026-08-22

**Authors:** Ahmed Nadeem

**Affiliations:** Department of Emergency Medicine, Stritch School of Medicine, Loyola University Medical Center, Maywood, Illinois, USA

**Keywords:** dental abscess, Ludwig's angina, facial cellulitis, facial swelling

## 1 Patient Presentation

**Figure 1:**
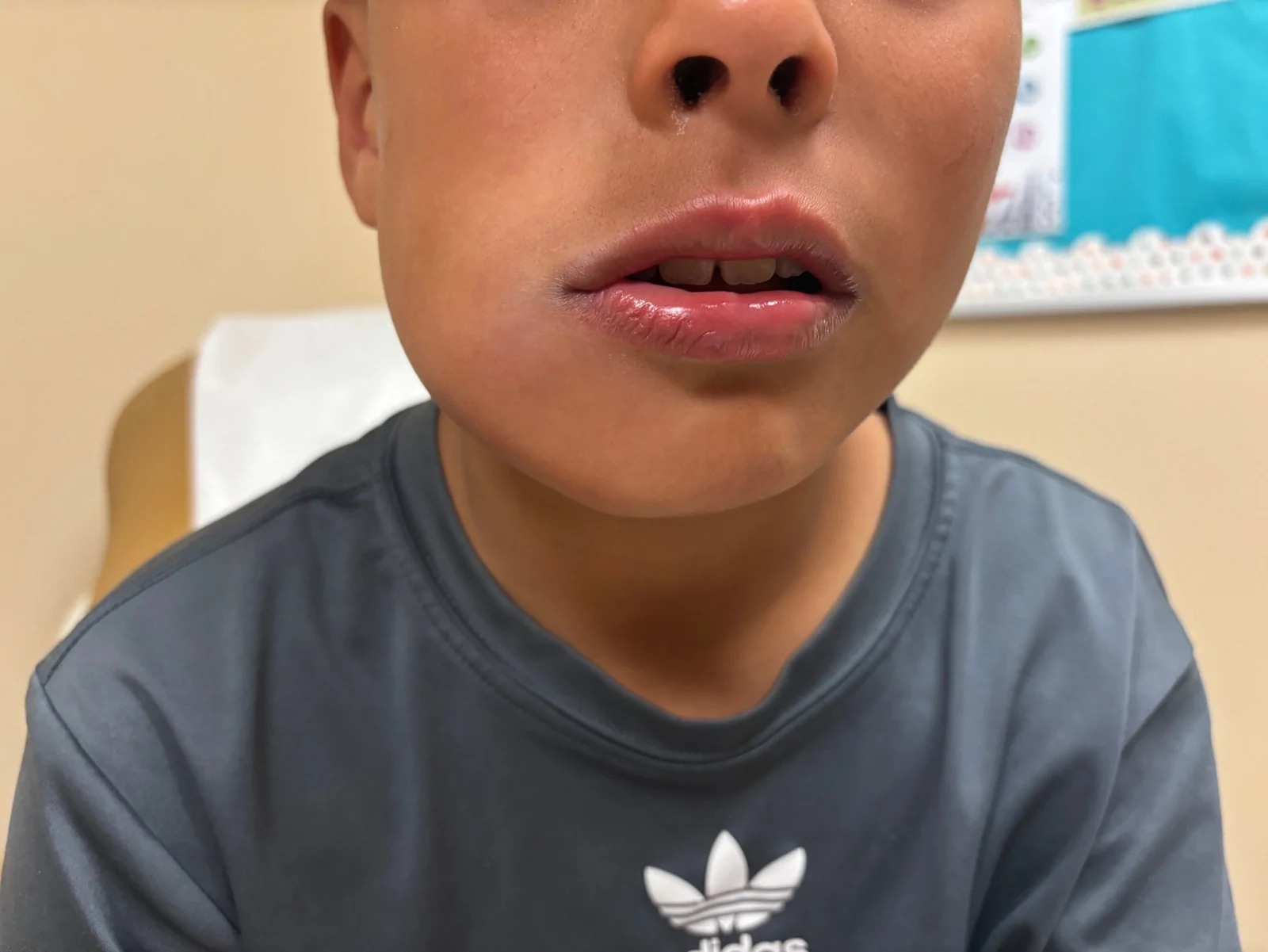
An 8-year-old boy with right facial swelling.

An 8-year-old boy presented to the immediate care with a 2-day history of right facial pain and swelling (Fig 1). He had a right lower molar cavity filled a year ago with a stainless-steel crown that chipped a month ago. Abnormal vital signs included a temperature of 38.1°C. He was alert, oriented, and hemodynamically stable with lower right facial swelling with tenderness, erythema, and warmth. The inferior border of the mandible could not be palpated, and there was swelling and erythema to the right mandibular buccal vestibule overlying a right 1st mandibular molar primary tooth. The tooth had a dental filling. There was mild trismus. He was transferred to the emergency department, where laboratory results yielded white blood cell (WBC) count of 16.9 ×10^9^/L and C-reactive protein of 64 mg/L. He had a computed tomography (CT) performed of the soft tissue neck with intravenous contrast.

## 2 Diagnosis

### 2.1 Subperiosteal Abscess With Facial Cellulitis

**Figure 2:**
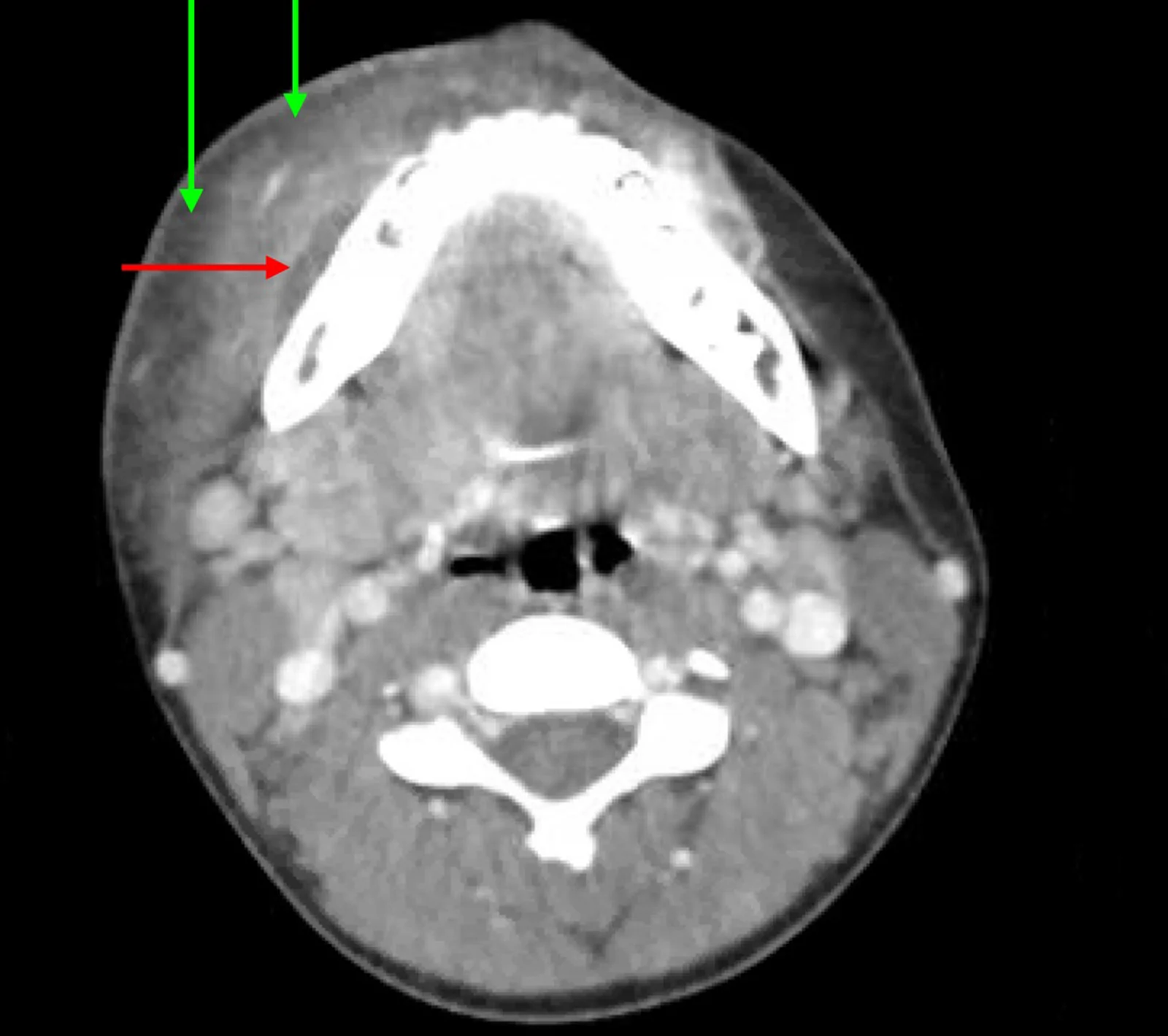
Axial view of CT scan—soft tissue of the neck. The red arrow denotes the subperiosteal abscess and the green arrow denotes the facial cellulitis. CT, computed tomography.

**Figure 3:**
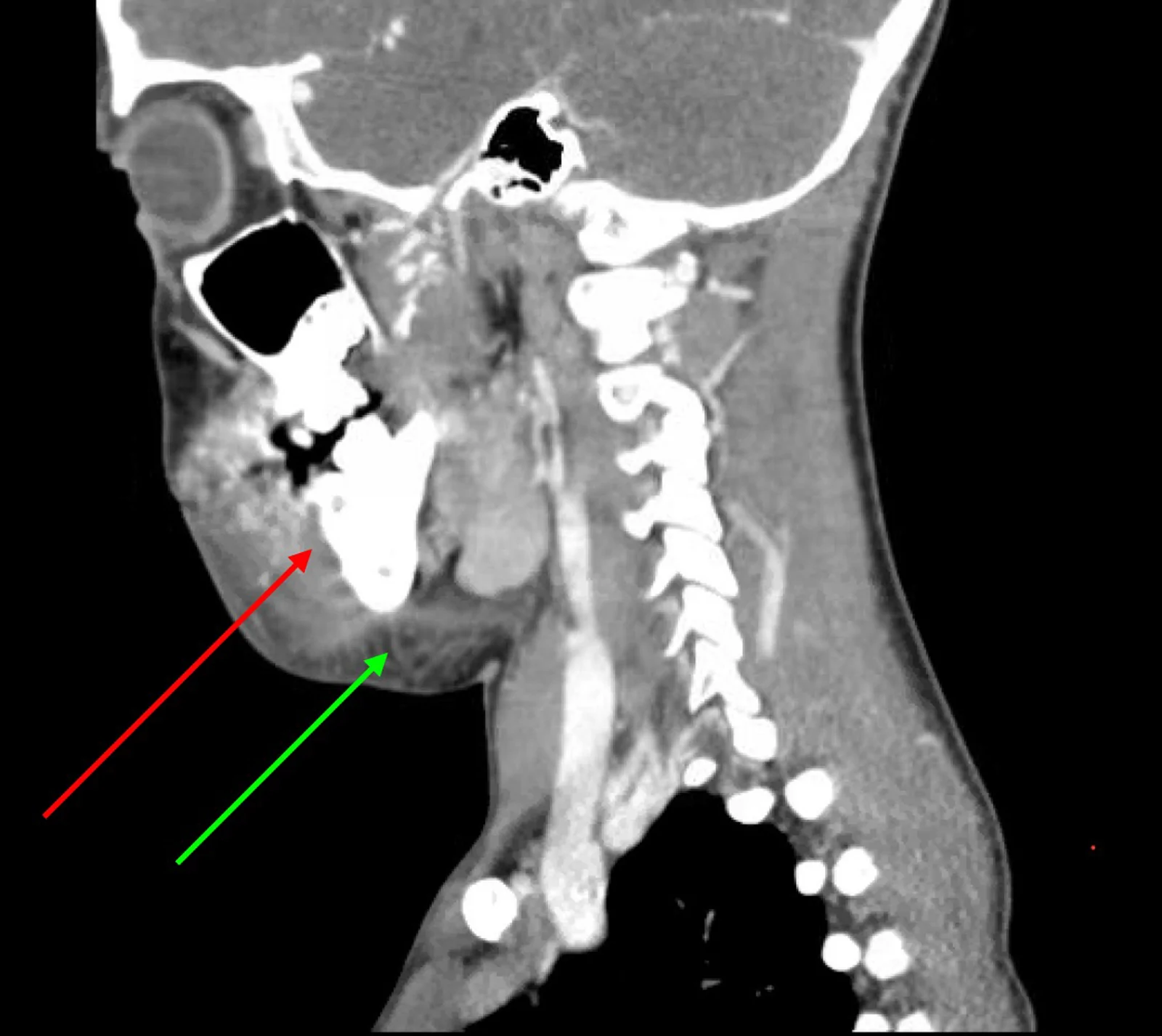
Sagittal view of CT scan—soft tissue of the neck. The red arrow denotes the subperiosteal abscess and the green arrow denotes the inferior part of the facial cellulitis. CT, computed tomography.

**Figure 4:**
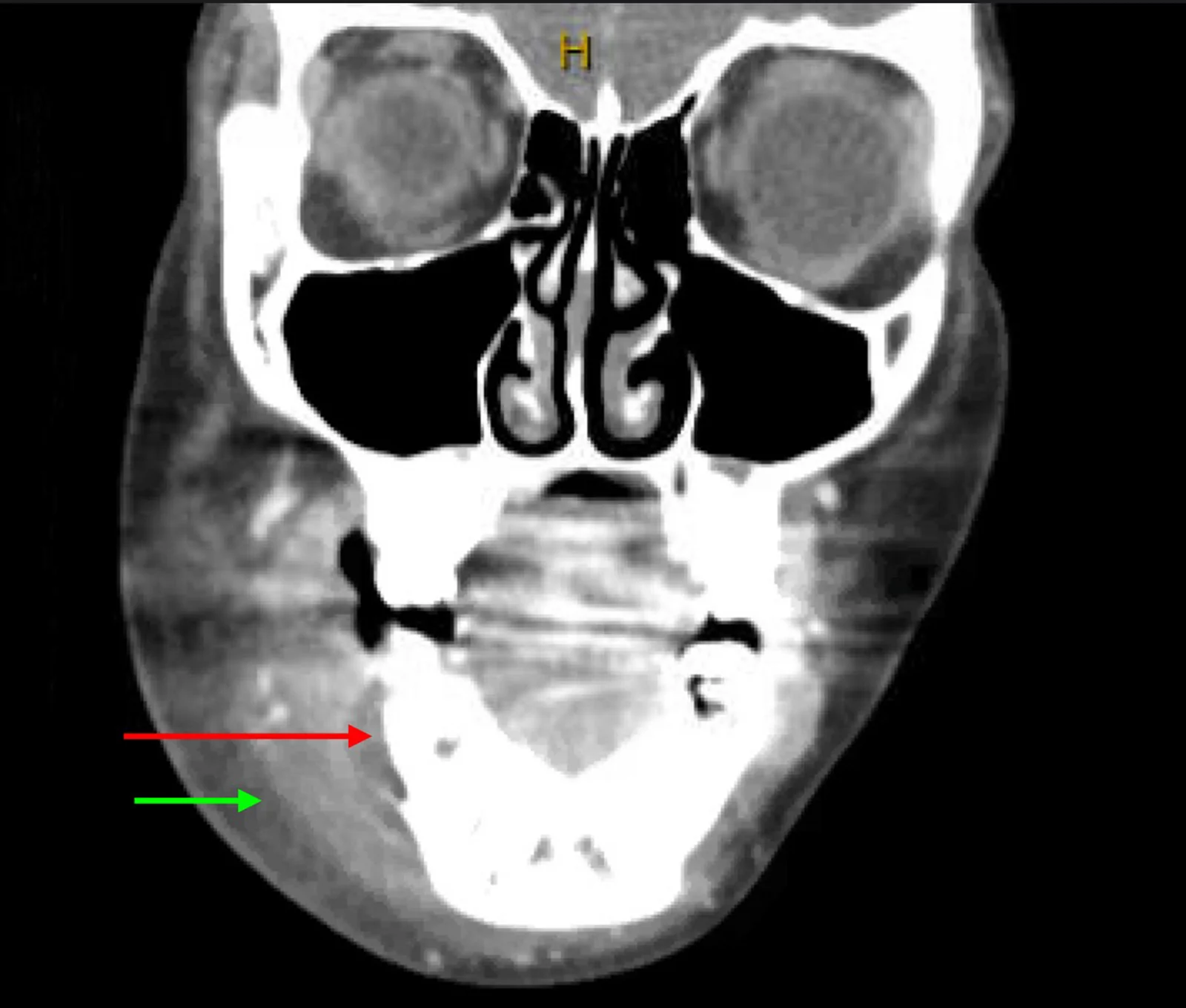
Coronal view of CT scan—soft tissue of the neck. The red arrow denotes the subperiosteal abscess, and the green arrow denotes subcutaneous reticulation and skin thickening compatible with right facial cellulitis and swelling. CT, computed tomography.

The CT demonstrated a subperiosteal abscess measuring 2.2 cm × 0.5 cm over the body of the right mandible next to the right 1st primary molar and soft tissue swelling over the right face and jaw with subcutaneous reticulation and skin thickening compatible with cellulitis (Figs 2, 3, and 4). Incision and drainage were performed by oral and maxillofacial surgery (OMFS). He was hospitalized, and ampicillin/sulbactam 50 mg/kg was administered. The culture grew *Streptococcus anginosus*. His WBC trended down, and he was discharged 3 days later with amoxicillin/clavulanate and OMFS follow-up.

Pediatric subperiosteal abscesses and facial cellulitis with mandibular source infections from a permanent tooth are a life-threatening diagnosis as they have less dense bone and more access to deep spaces of the head and neck than adults. Facial cellulitis indicates that the infection has breached the periosteum and extended into the soft tissues.^1^ The infection can extend to the sublingual and submandibular spaces and progress to Ludwig's angina, leading to airway compromise.^2^ It can lead to descending necrotizing mediastinitis through the parapharyngeal and retropharyngeal spaces. It can also lead to osteomyelitis of the mandible and sepsis.^1^ It requires immediate airway evaluation, incision, drainage, and broad-spectrum intravenous antibiotics.

## Funding and Support

By *JACEP Open* policy, all authors are required to disclose any and all commercial, financial, and other relationships in any way related to the subject of this article as per ICMJE conflict of interest guidelines (see www.icmje.org). The author has stated that no such relationships exist.

## Conflict of Interest

The author has affirmed they have no conflicts of interest to declare.
